# Predictive modeling of wide-shallow RC beams shear strength considering stirrups effect using (FEM-ML) approach

**DOI:** 10.1038/s41598-024-62532-y

**Published:** 2024-05-31

**Authors:** Ahmed A. Soliman, Dina M. Mansour, Ayman H. Khalil, Ahmed Ebid

**Affiliations:** 1 Structural Engineering & Construction Management Department, Faculty of Engineering & Technology, Future University in Egypt, New Cairo, Egypt; 2 Structural Engineering Department, Faculty of Engineering, Ain Shams University, Cairo, Egypt

**Keywords:** Wide beams, Shallow beams, Machine learning, Artificial neural networks, Shear capacity, Genetic programing, Evolutionary polynomial regression, Civil engineering, Software

## Abstract

This paper presents an analysis and prediction of the shear strength of wide-shallow reinforced concrete beams, utilizing Finite Element Analysis (FEA) and machine learning techniques. The methodology involves validating a detailed Finite Element Model (FEM) against experimental results, conducting a parametric study, and developing three Machine Learning prediction equations. The FEM captures concrete and steel behaviors, including cracking and crushing for concrete and linear isotropic properties for steel reinforcement. Loading and boundary conditions are defined for accuracy and validated against 13 experimental specimens, exhibiting a maximum 8% and 12% difference in loads and deflections, respectively. A parametric study generates a dataset of 77 wide beam configurations, exploring variations in beam widths, concrete strengths, compression rebars, and shear reinforcement. This dataset is used to develop machine learning models, including “Genetic Programming (GP)”, “Evolutionary Polynomial Regression (EPR)”, and “Artificial Neural Network (ANN)”. Comparative analysis reveals GP and EPR models with over 95% correlation, while the ANN model outperforms with 99% accuracy. Sensitivity analysis underscores the significant influence of concrete strength and beam aspect ratio on shear strength. In conclusion, the study demonstrates the potential of FEA and machine learning models to predict shear strength in wide-shallow reinforced concrete beams, providing valuable insights for architectural design and engineering practices and emphasizing the role of concrete strength and beam geometry in shear behavior.

## Introduction

Wide-shallow beams, renowned for their architectural elegance and structural efficiency, have been a prominent choice in architectural applications. Despite their popularity, understanding the intricate shear behavior of these beams has been a focal point of extensive research. Various factors, such as the longitudinal spacing between stirrups, stirrup leg spacing, and shear reinforcement ratio, significantly influence their shear capacity. Earlier code provisions overlooked the role of shear reinforcement and stirrups in wide beams, despite the initiation of research on their shear behavior several decades ago. Initial definitions by researchers categorized wide beams as those with widths twice their depths^1^. However, Soliman et al. in 2023^2,3^ highlighted gaps in understanding wide beam behavior, inspiring a gradual exploration of numerous parameters and variables. Research, often experimental and complemented by finite element analysis, has progressively filled these knowledge voids, enhancing the comprehension of the complex shear behavior of wide-shallow beams.

The exploration of numerical investigations for concrete members has a longstanding history, offering a pathway to attain highly accurate results contingent on precise input data. Many studies in this field incorporate experimental programs to validate Finite Element Models (FEM) through comparisons with real-world results, enabling adjustments to parameters for additional insights. Throughout various research programs, ANSYS^4^ and ABAQUS^5^ have consistently emerged as the most widely employed finite element programs for this purpose. These programs' versatility enables researchers to simulate and analyze complex phenomena, ensuring a thorough understanding of concrete member behavior. The combination of numerical simulations and experimental validation contributes to refining Finite Element Models, enhancing their reliability and applicability in addressing diverse challenges related to concrete structures.

Numerical investigations in the domain of concrete structures have been integral to advancing the accuracy of Finite Element Models (FEM). Researchers such as Bach Luu et al.^6^ have engaged in FEM validation against experimental results, noting an 11% model error in their study of two concrete beams. The significance of this validation process lies in its potential to refine models and improve predictions. Additionally, their parametric study conducted explored the impact of rebar diameters on crack width, aiming to optimize concrete performance. The use of FEM, as exemplified by these studies, continues to be a crucial tool for understanding and predicting the behavior of concrete structures.

Wani et al.^7^ contributed to the field by successfully predicting the mechanical behavior of reinforced concrete beams through the application of FEM. Their model aligned well with experimental findings, providing valuable insights into failure mechanisms and ultimate loads. Meanwhile, Derseh et al.^8^ delved into finite element analysis for both conventional and specially reinforced concrete beams subjected to impact loads. While acknowledging a low margin of error in their FEM results, the authors highlighted the necessity for future studies to consider the resilience of impact loading, underscoring the importance of continual refinement in numerical modeling techniques for concrete structures.

In their 2021 study, Tambusay et al.^9^ conducted a thorough comparison of experimental and Finite Element Method (FEM) results for twelve reinforced concrete beams, revealing the FEM's significant accuracy of 94% in shear failure analysis, particularly with the smeared crack approach. In a similar study, Tahenni et al.^10^ tested sixteen reinforced concrete beams, validating the FEM with a maximum difference of 14% between experimental and simulated results. However, extra caution was given for the potential overestimation of the contribution of transverse reinforcement in the FEM, emphasizing the ongoing challenges of accurately representing real-world behavior in numerical models. The critical role of FEM in these studies showcases its utility in advancing the understanding of concrete structures and their complex behaviors. Solahuddin and Yahaya^11^ conducted an extensive investigation into Finite Element Analysis (FEA) studies related to Fiber Reinforced Polymer (FRP) concrete beams. Their research comprehensively addressed critical issues in this domain, focusing on material non-linearity, separation failure, and debonding. In a parallel effort, Shahrbijari et al.^12^ developed a dedicated Finite Element Model (FEM) for fiber-reinforced concrete beams, specifically evaluating the global resistance safety factor. The authors recommended considering a higher safety factor than the standard, emphasizing the importance of meticulous safety assessments in the design and analysis of structures incorporating fiber reinforcement.

Recent advancements in Machine Learning (ML) have sparked new research opportunities in the field of concrete elements. Several recent studies have utilized machine learning techniques to predict the shear capacity and mode of failure of reinforced concrete beams^13–16^. These studies collectively suggest that machine learning stands out as a promising and effective approach for forecasting the behavior of concrete elements, marking a noteworthy shift in the methodologies applied to concrete structural analysis and design^17^.

Capitalizing on the capabilities of Machine Learning, Ebid and Deifalla (2021–2022)^18,19^ conducted pioneering research in the realm of concrete structural analysis. Their work focused on developing equations using Genetic Programming (GP) and Evolutionary Polynomial Regression (EPR) techniques to predict the shear capacities of various types of concrete beams. Notably, both GP and EPR methods demonstrated remarkable accuracy in predicting shear capacities, showcasing the potential of these Machine Learning techniques in advancing predictive capabilities in the field of concrete structural behavior.

Extensive experimental testing has been conducted on wide, shallow reinforced concrete beams, with numerous studies contributing valuable insights into their behavior and performance^20–22^. A research initiative aimed to address gaps in existing codes of practice that might not comprehensively cover the unique characteristics of wide beams^23^. Some studies even conceptualized the behavior of these wide beams as akin to one-way slabs, offering a perspective that enhances the grasp of their structural response^24,25^. Additionally, efforts have been made to incorporate considerations for shear reinforcement specifically tailored to the distinctive features of wide beams, reflecting the ongoing endeavors to refine and augment provisions related to these structural elements.

Soliman et al.^26,27^ conducted a comprehensive investigation into the shear capacity of seven wide-shallow beam specimens, exploring various influencing factors such as aspect ratio, concrete compressive strength, compression rebar ratio, longitudinal spacing between rebars, transverse spacing between rebars, and shear reinforcement. Their findings revealed that the ultimate shear strength of the specimens exhibited a decreasing trend with an increase in the aspect ratio, varying from 1.66 to 5. Moreover, the shear capacity of the beams demonstrated an upward trajectory with an increase in compressive strength, while the impact of compression rebar on shear capacity was comparatively modest. Notably, the configuration of stirrups was identified as a factor with a discernible impact on shear capacity, with shear reinforcement ratio emerging as the most influential parameter in determining the overall shear strength of the wide-shallow beams.

As discussed earlier, various machine learning techniques have been employed by previous researchers to formulate equations for predicting shear capacity values in reinforced concrete beams. However, none of these studies exclusively addressed the unique characteristics of wide-shallow reinforced concrete (RC) beams, which are discussed in this paper.

## Objectives

The primary goal of this research is to develop predictive models for the shear capacity of wide or shallow RC beams, including the effect of shear reinforcement amount and arrangement, aspect ratio, concrete strength, and compression rebars. Three machine learning (ML) prediction models were developed using “Genetic Programming (GP)”, “Evolutionary Polynomial Regression (EPR)”, and “Artificial Neural Network (ANN)” techniques. These (ML) models are intended to serve as valuable tools for designers and researchers, providing insights and predictions related to the behavior of wide, shallow reinforced concrete beams. The predictions derived from these techniques offer a systematic and efficient means to explore the performance of such beams under varying conditions and parameters considered in the study.

## Methodology

To accomplish the objectives, the research employs a systematic approach that involves the validation of a Finite Element Model (FEM) against results obtained from the earlier stages of the experimental program^26,27^. This validation process serves as a crucial step in ensuring the accuracy and reliability of the FEM. Subsequently, a comprehensive comparison is conducted between the FEM results and the outcomes of the experimental program. Following this comparison, a thorough parametric study is undertaken based on the validated model, aiming to explore the impact of the various parameters considered in this study. The research meticulously examines the discrepancies, errors, and accuracies between the FEM predictions and the experimental data. Building upon this foundation, the study culminates in the development of three Machine Learning (ML) prediction models. These equations, grounded in “Genetic Programming (GP)”, “Evolutionary Polynomial Regression (EPR)”, and “Artificial Neural Network (ANN)” techniques, play a pivotal role in advancing the predictive capabilities related to the behavior of the beams under investigation.

### Finite element model

All the details of the steel and concrete used in the developed model that are applied in ANSYS are presented in this section.

#### Concrete

Concrete was modeled using SOLID65 elements, having eight nodes, and three degrees of freedom and capabilities for both cracking and crushing (28).

**Figure 1:**
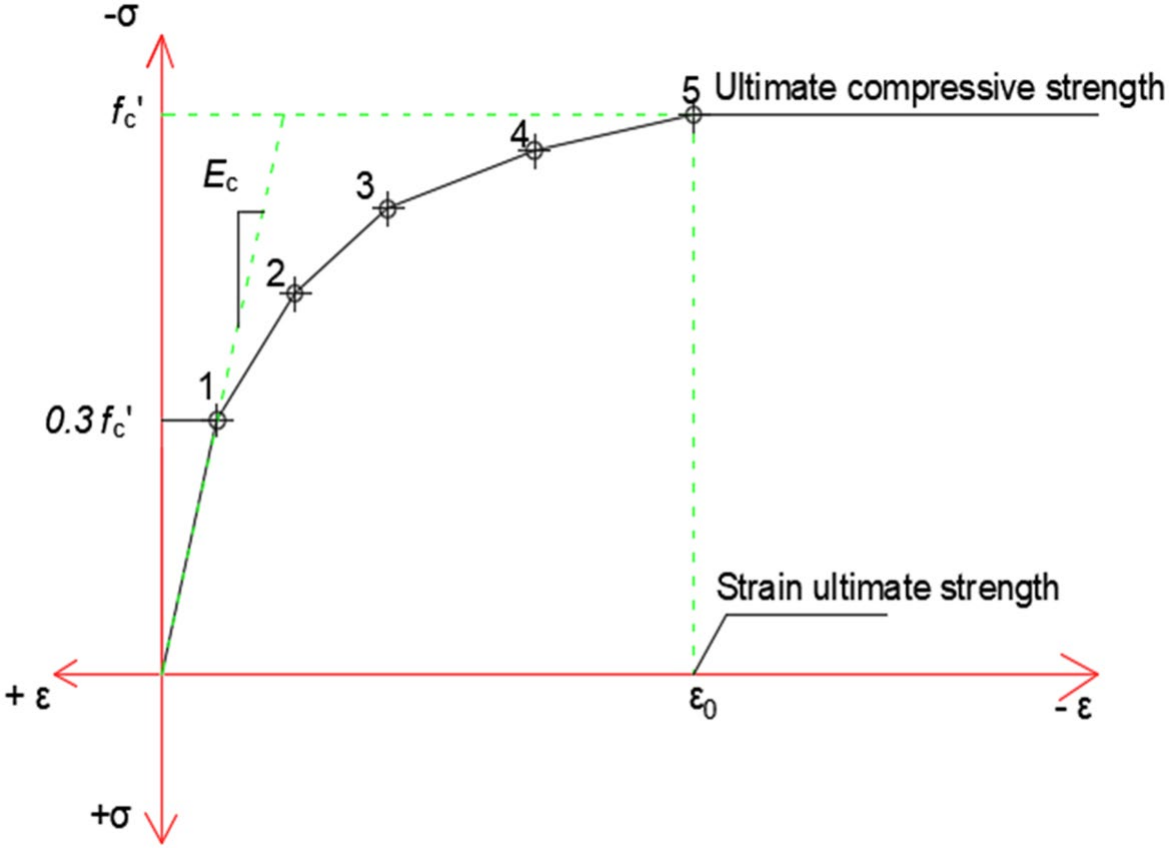
Simplified uniaxial stress–strain curve for concrete^29^.

Three different concrete mixtures were modeled in the FEM program to get three different characteristic strengths (Fc’ = 22 MPa, 31.2 MPa, and 37 MPa). The three mixtures had the same Poisson ratio = 0.2, open shear coefficient = 0.2, and closed shear coefficient = 0.8. Uniaxial cracking and uniaxial crushing depended on each mixture. Also, stress strain curves were entered into the program manually, as presented in Fig. 1. Point No. 1 at 0.30 f.’c is calculated for the stress–strain relationship of the concrete in the linear range. Points No. 2, 3, 4, and 5 are at ɛ_0_ and f’c. An assumption was made in this study of perfectly plastic behavior after point No.5, and all were calculated from Eqs. (1–3).

1 $$f = \frac{Ec . \upvarepsilon }{{1 + \left( {\frac{\upvarepsilon }{\upvarepsilon o}} \right)}}$$

2 $$\upvarepsilon o = \frac{{2f^{{^{\prime } }} c}}{Ec}$$

3 $$\text{Ec}= \frac{f}{\upvarepsilon }$$

where

f = stress at any strain ε.

ε = strain at stress f.

ε_o_ = strain at ultimate compressive stress fc ‘

#### Steel

Both longitudinal steel reinforcement (tension and compression) and shear reinforcement were modeled using LINK180 elements, it’s a 3D spar element with three degrees of freedom and a uniaxial tension–compression member with the capability to input a temperature load as a body load^28^.

**Figure 2:**
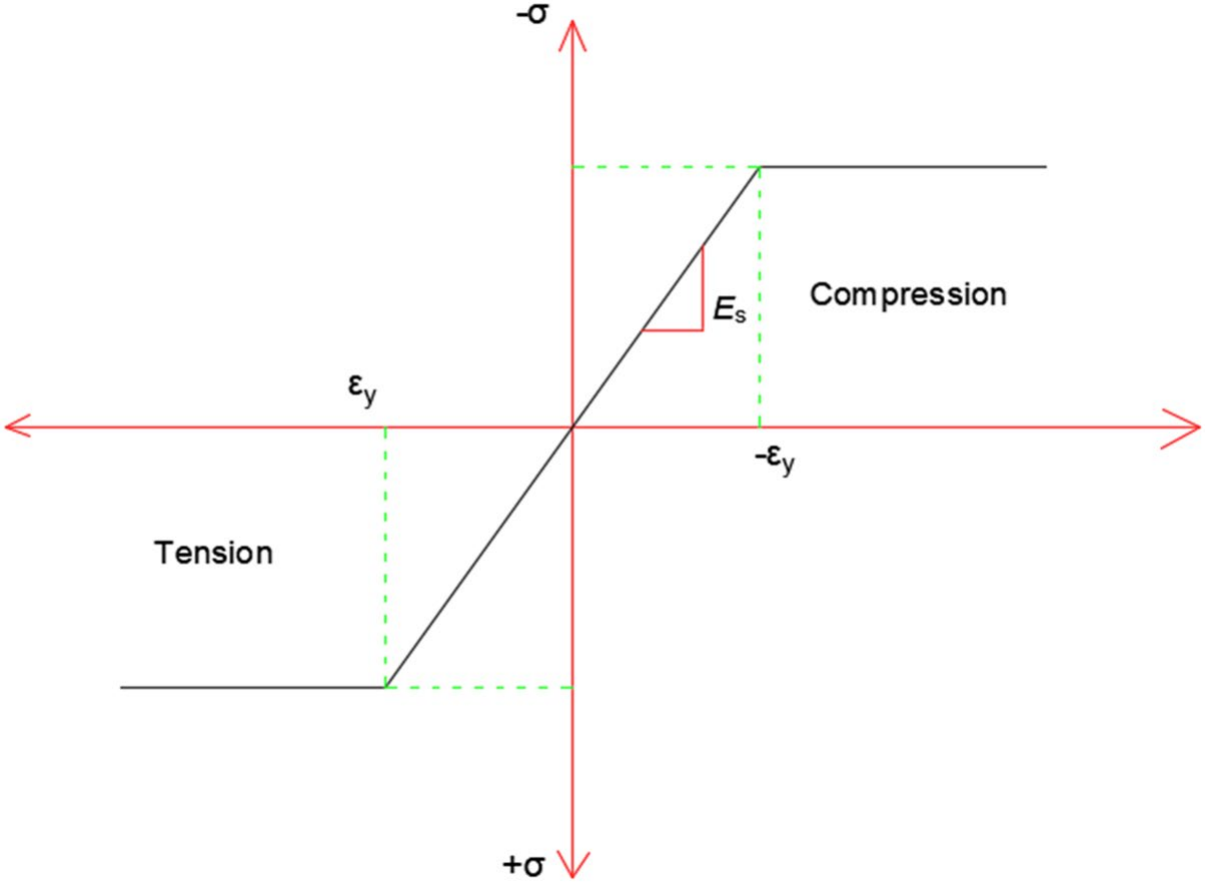
Simplified uniaxial stress–strain curve for steel^29^.

Two types of steel reinforcement were defined as high tensile and mild steel with two different moduli of elasticity (Es = 2.1 × 10^7^ t/m^2^ and 2 × 10^7^ t/m^2^, respectively) and they had the same Poisson ratio of 0.3 as a linear isotropic property. Also, both types of reinforcement had a bilinear property (yield strength = 240 MPa and 400 MPa, respectively). All the previously mentioned properties are presented in Fig. 2.

#### Loading and boundary conditions

**Figure 3:**
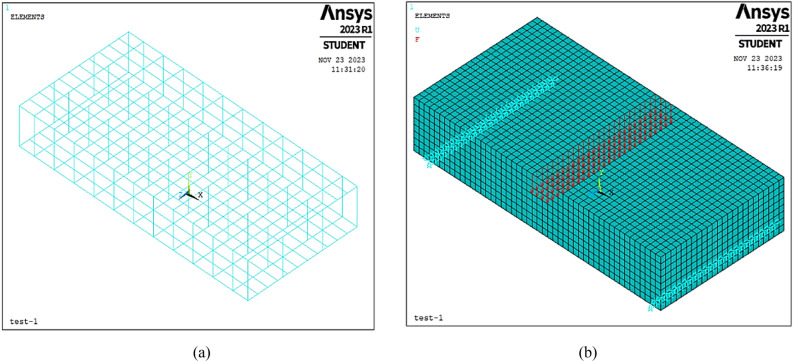
The developed FEM model, (**a**) Steel arrangement, (**b**) Meshing and boundary conditions.

**Figure 4:**
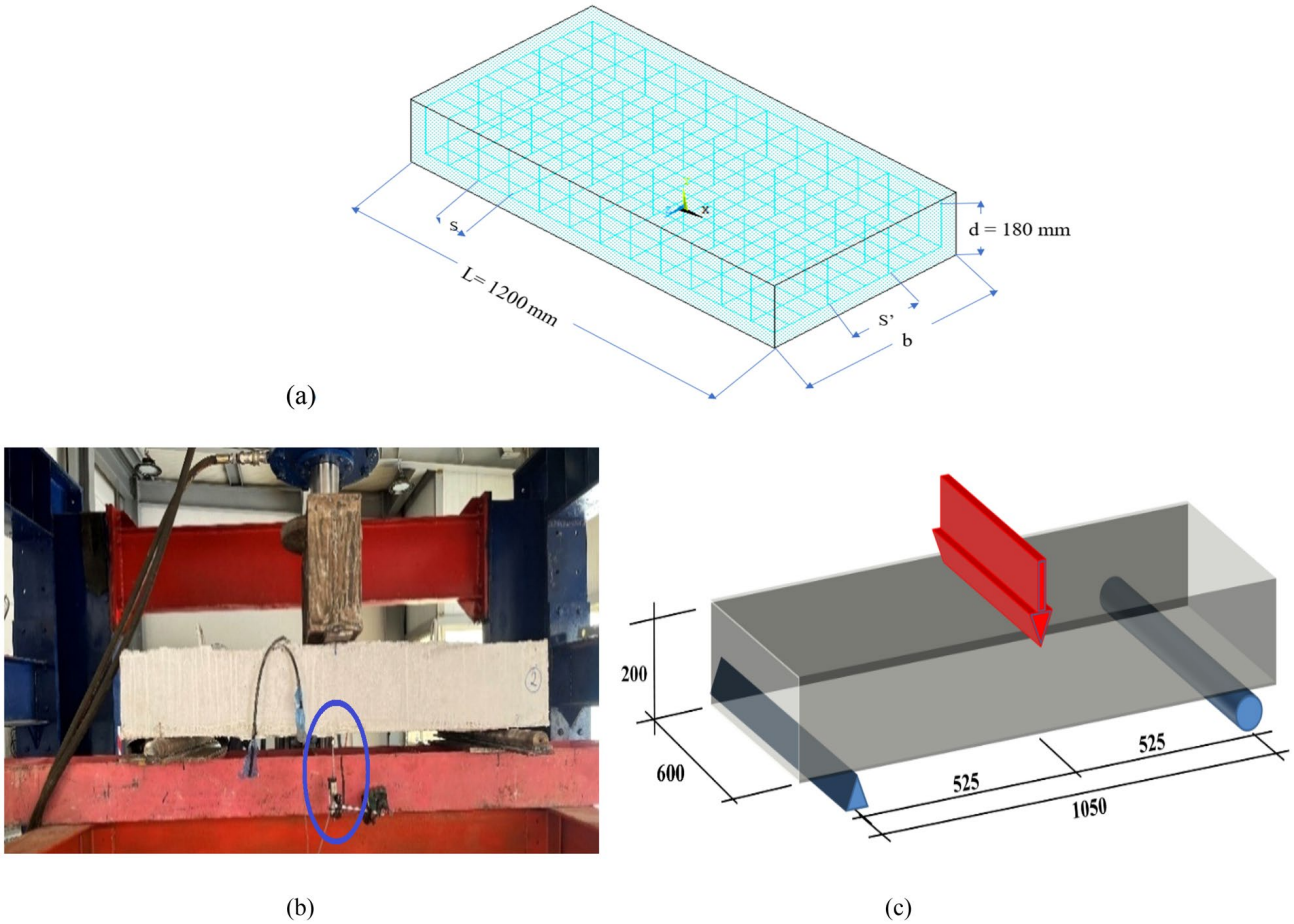
(**a**) The details and definition of the parameters. (**b**), (**c**) test setup of the experimental program^26,27^.

To achieve an appropriate degree of accurcy, a sensitivity analysis was made, and a mesh of 2.5 cm was used in meshing each specimen, as shown in Fig. 3a and b. The specimens were restrained at the lower face in the short direction, 7.5 cm along the X-axis from the edge of the specimen from both sides of the specimen. So, each specimen is restrained at two sets of nodes to simulate the steel rods from the experimental phase, the first set at X and Y directions and the other at X only. Loading was modelled as forces in the Y direction of the specimens placed on the lines of nodes at the center of the specimens to simulate the experimental setup as shown in Fig. 4 same as what went down at the experimental phase.

#### Validation of the FEM Model

The FEM model was validated using results of previous research programs^26,27^. The criteria for selecting parameters in the experimental study were based on their significant influence on the shear capacity of wide-shallow reinforced concrete beams. These parameters included the aspect ratio (b/d) of the beams, the longitudinal spacing of stirrups to depth ratio (S/d), the transverse spacing of stirrups to depth ratio (S^′^\d), the concrete characteristic strength (Fc^′^), the shear reinforcement strength (µ fys), and the compression reinforcement (ρ^′^). The goal was to systematically investigate the impact of these key factors on the behavior and total shear capacity of the specimens, providing a comprehensive understanding of the shear performance of wide-shallow beams under various conditions. The program consisted of 13 specimens of reinforced concrete shallow, wide beams with dimensions of 600 × 1200 × 200 mm as shown in Fig. 4a. All the specimens had the same longitudinal reinforcement to the cross-section ratio (ρ = As/bd = 2.83%). The specimens were designed to fail at shear under a three-point loading test, as shown in Fig. 4b and c. The details of the tested specimens are presented in Table 1.

**Table 1:** The configurations of all tested samples^26,27^. The studied parameters for each specimen are expressed in bold.

Beam ID	(b/d)	(S/d)	(S'/d)	Fc^′^ (Mpa)	µ fys (Mpa)	ρ^′^ = (As^′^/(b*d)) (%)
B1	3.33	0.55	1.11	31.2	0.77	0.58
B2	**1.67**	0.55	1.11	31.2	0.77	0.58
B3	**5.00**	0.55	1.11	31.2	0.77	0.58
B4	3.33	**0.28**	1.11	31.2	0.77	0.58
B5	3.33	**1.11**	1.11	31.2	0.77	0.58
B6	3.33	0.55	**0.67**	31.2	0.77	0.58
B7	3.33	0.55	**3.33**	31.2	0.77	0.58
B8	3.33	0.55	1.11	**22.0**	0.77	0.58
B9	3.33	0.55	1.11	**37.0**	0.77	0.58
B10	3.33	0.55	1.11	31.2	**0.44**	0.58
B11	3.33	0.55	1.11	31.2	**1.21**	0.58
B12	3.33	0.55	1.11	31.2	0.77	**1.42**
B13	3.33	0.55	1.11	31.2	0.77	**2.36**

**Figure 5:**
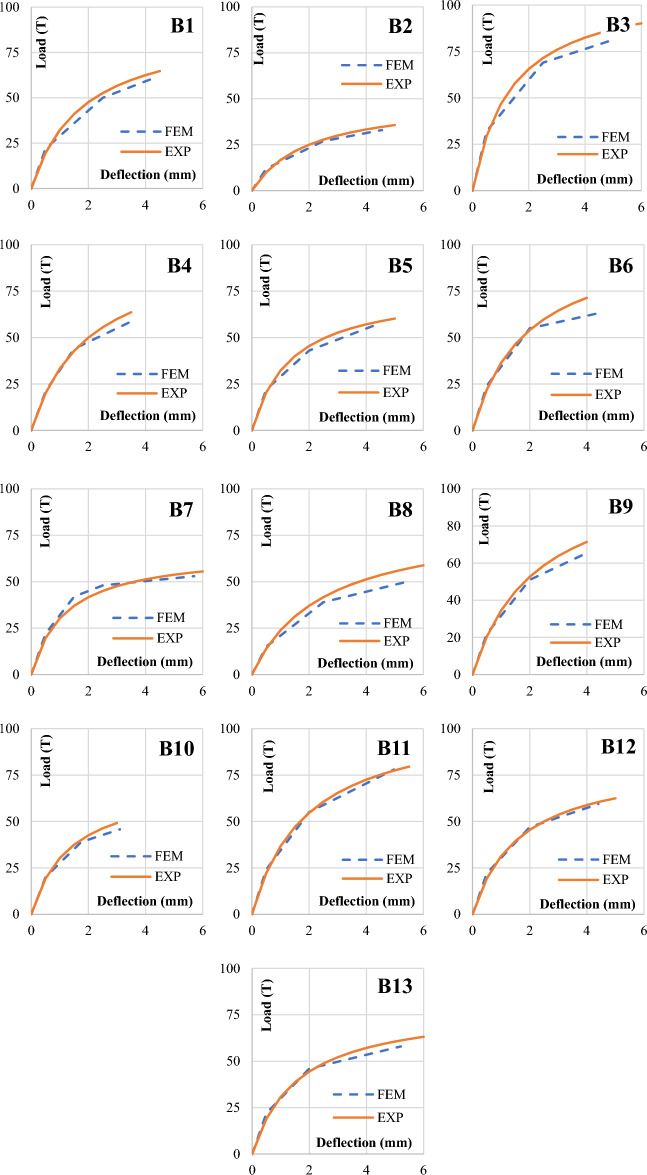
Comparison between load–deflection curves of both experimental and FEM results for each tested specimen.

Each tested specimen had been modeled using the developed FEM model and was compared to each load–deflection curve of each specimen, as shown in Fig. 5. The results showed differences between the outputs in loads and deflections of a maximum of 8% and 12%, respectively.

### Parametric study

The validated FEM model obtained from the previous section was used to develop a parametric study. Models were designed to simulate a wide range of reinforced concrete wide beams while changing the parameters used through the experimental results to obtain a reliable dataset for further analysis.

#### Developed database

Utilizing a validated EFM model, 77 records were systematically generated for wide beams characterized by identical span, depth, tension rebars, and loading setup (3-point bending test) as presented in the Appendix. The variation in the records stemmed from differences in beam widths, concrete strengths, compression rebars, and the configuration of shear reinforcement, including both the amount and spacing. This comprehensive approach allowed for a nuanced exploration of the influence of diverse parameters on the behavior of wide beams, offering valuable insights into their structural performance under various conditions.

**Figure 6:**
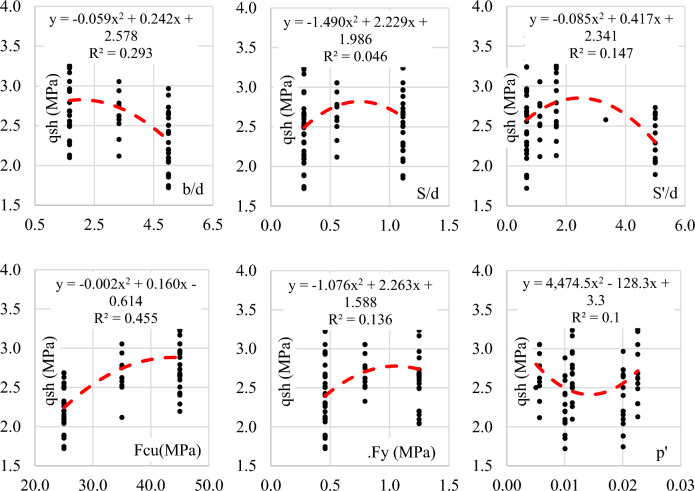
Relationship between the different parameters and the total shear capacity (qsh).

**Figure 7:**
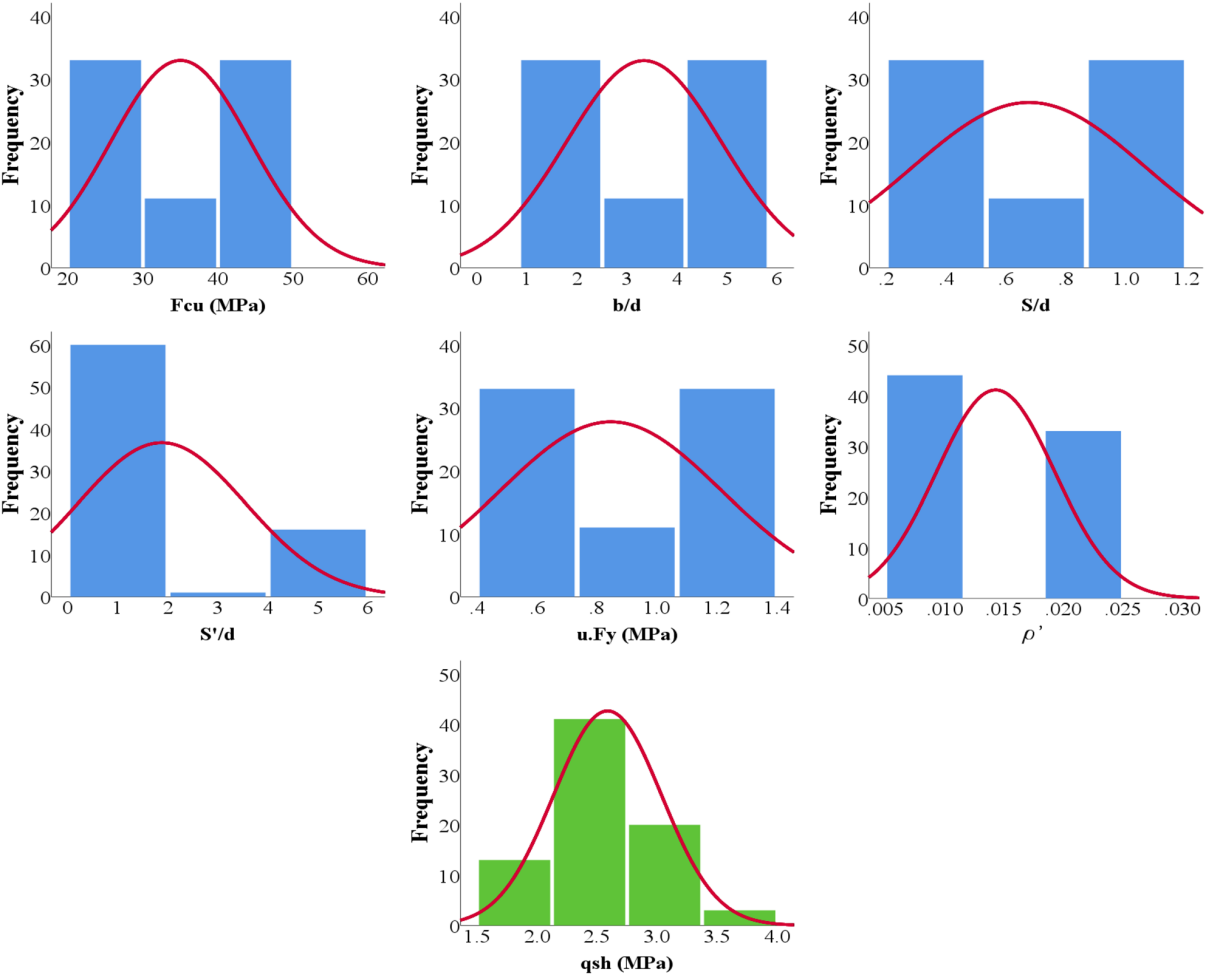
Distribution histograms for inputs (in blue) and outputs (in green).

The generated records were carefully organized into a training set, comprising 64 records, and a validation set, consisting of 13 records. The statistical characteristics and the interrelationship between variables were systematically summarized and presented in Tables 2 and 3. The Pearson correlation matrix was employed to delineate the dependencies among the various parameters. Additionally, Figs. 6 and 7 provided insightful visual representations, illustrating the relationships between inputs and outputs, along with histograms depicting the distributions of both inputs and outputs. This rigorous organization and analysis of the generated records enhances the comprehensibility and utility of the dataset, offering a valuable resource for further investigations into the behavior of wide beams.

**Table 2:** Statistical analysis of developed database.

	Fcu	b/d	S/d	S'/d	μ.Fy	ρ^′^	qsh
	MPa	–	–	–	MPa	–	MPa
	Training dataset						
Max	45.00	5.00	1.11	5.00	1.25	0.02	3.62
Min	25.00	1.67	0.28	0.67	0.46	0.01	1.72
Avg	34.83	3.36	0.63	1.96	0.84	0.01	2.56
SD	9.22	1.54	0.39	1.74	0.37	0.01	0.42
Var	0.26	0.46	0.61	0.89	0.44	0.43	0.16
	Validation dataset						
Max	45.00	5.00	1.11	5.00	1.25	0.02	3.68
Min	25.00	1.67	0.28	0.67	0.46	0.01	1.85
Avg	35.59	3.24	0.82	1.55	0.87	0.01	2.70
SD	9.37	1.56	0.36	1.33	0.37	0.01	0.52
Var	0.26	0.48	0.44	0.86	0.43	0.43	0.19

**Table 3:**
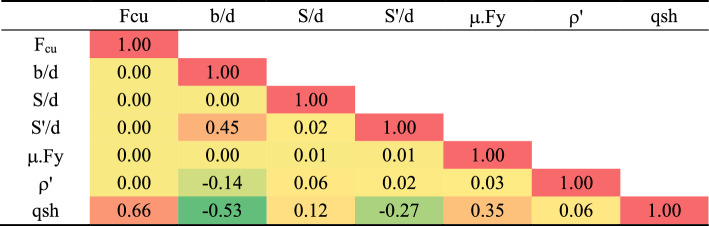
Pearson correlation matrix.

#### Analysis of the outputs

**Figure 8:**
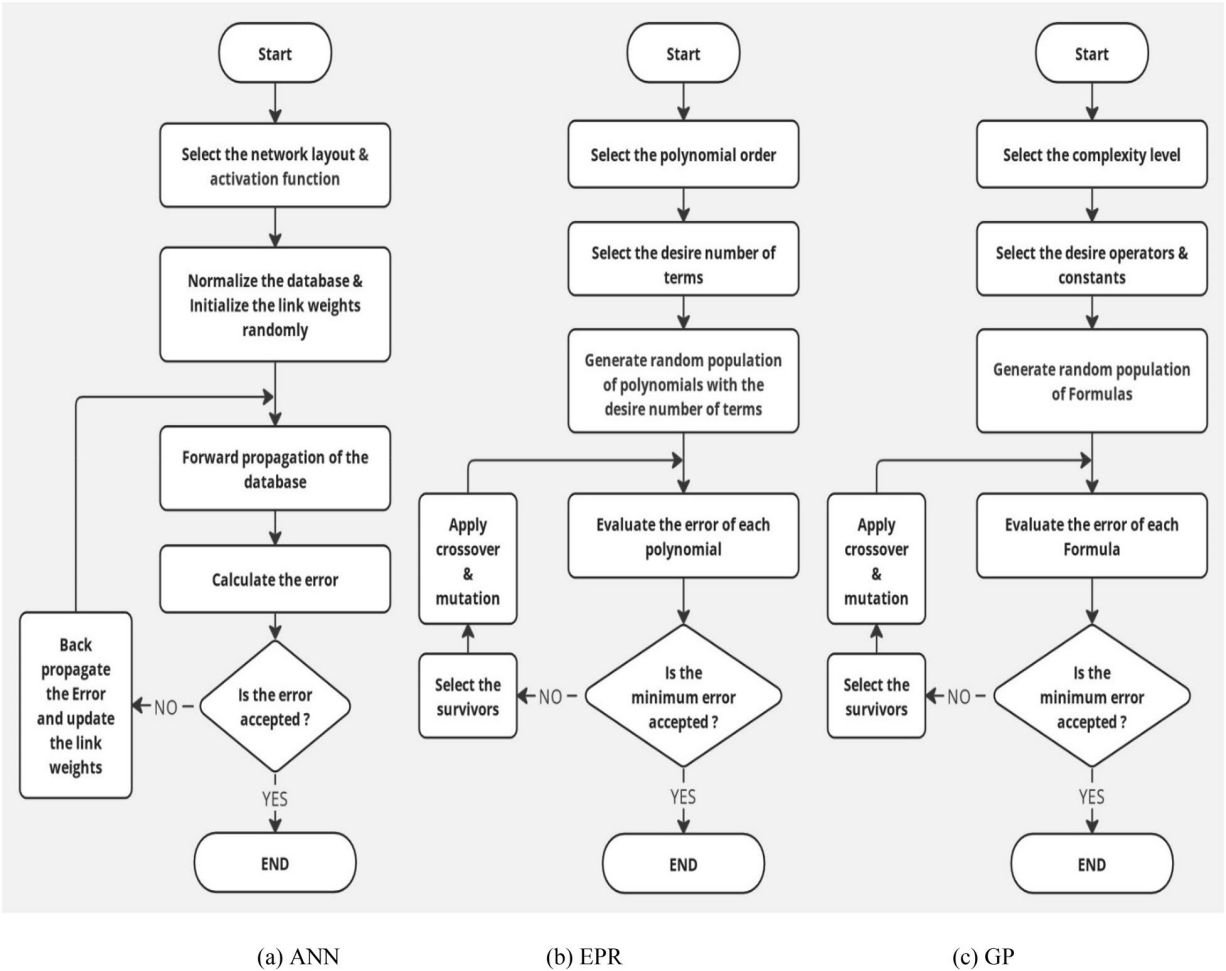
Flowcharts for the used (ML) regression techniques.

Three distinct Machine Learning (ML) regression techniques, namely "Artificial Neural Network" (ANN), "Genetic Programming" (GP), and "Evolutionary Polynomial Regression" (EPR), were employed to forecast the total shear strength (qsh) of RC beams using the meticulously generated database. The flowcharts for these applied techniques are elucidated in Fig. 8. Each of the three models was strategically employed to predict (qsh) based on material strength parameters (Fcu, μ.Fy), geometrical configuration (b/d), and rebar arrangement details (S/d, S^′^/d). The ensuing section delves into an in-depth exploration of the outcomes derived from each model, providing a comprehensive analysis of their accuracies.

To evaluate the effectiveness of the developed models, a meticulous assessment was conducted by scrutinizing the misclassified cases (error %) between the predicted and observed (qsh). This detailed analysis offers valuable perceptions into the performance and reliability of each ML regression technique in predicting the shear strength of RC beams, contributing to a broader awareness of the applicability of these models in structural engineering research and design.

A preliminary sensitivity analysis was meticulously conducted on the comprehensive database to assess the influence of each input variable on the total shear strength (qsh) values. Employing the "single variable per time" technique, the "Sensitivity Index" (SI) for each input was determined using the formula proposed by Hoffman and Gardener in 1983^30^ as follows:

4 $$SI \left({X}_{n}\right)= \frac{Y\left({X}_{max}\right)-Y\left({X}_{min}\right)}{Y\left({X}_{max}\right)}$$

Consequently, the obtained sensitivity indices are documented as follows: 0.27 for Fcu, 0.25 for b/d, 0.10 for S/d, 0.10 for S^′^/d, 0.15 for μ.Fy, and 0.15 for ρ^′^. These sensitivity indices provide a quantitative measure of the impact of each input parameter on the shear strength prediction, aiding in highlighting the relative importance of different factors in the model.

Moreover, it is essential to interpret the sensitivity indices in the context of the defined range; a sensitivity index of 1.0 signifies complete sensitivity, while a value below 0.01 indicates that the model is relatively insensitive to changes in the corresponding parameter. This demonstrates the significance of each input variable, guiding researchers and engineers in prioritizing parameters that significantly influence the shear strength predictions. The detailed sensitivity analysis contributes to the robustness and reliability of the developed models, enhancing their utility in practical applications within the realm of structural engineering.

### Proposed prediction models using machine learning techniques

Evolutionary Polynomial Regression (EPR), Genetic Programming (GP), and Artificial Neural Network (ANN) emerge as superior choices for predicting shear strengths in wide-shallow reinforced concrete beams within this study's context. Their collective efficacy surpasses that of other machine learning techniques for several reasons.

EPR and GP, as symbolic regression methods, excel at capturing complex relationships inherent in structural datasets. Their ability to evolve mathematical expressions provides a nuanced understanding of the intricate interdependencies among various parameters, offering more accurate predictions for the shear capacities of wide-shallow beams.

On the other hand, ANN's strength lies in its adaptability and capacity for automatic feature learning. The hierarchical structure of neural networks allows them to discern subtle correlations and adapt to variations in input parameters, making them particularly effective in addressing the multifaceted and non-linear nature of shear behavior in concrete beams.

The collective superiority of EPR, GP, and ANN over other machine learning techniques can be attributed to their unique strengths—EPR and GP's prowess in symbolic regression and ANN's adaptability and feature learning capabilities. These qualities enable them to navigate the complexities of wide-shallow reinforced concrete beams more effectively than alternative methods.

#### (GP) Model

Genetic Programming (GP) emerges as a powerful computational tool with specific applications in civil engineering. In this domain, GP is utilized to evolve mathematical expressions that capture intricate relationships among structural parameters, material properties, and other influential factors. Employing principles inspired by natural selection, GP iteratively refines and evolves mathematical models to fit empirical data. Civil engineers leverage GP to develop predictive models for structural performance, aiding in the optimization of design solutions and decision-making processes. The adaptability and efficiency of GP makes it a valuable tool for addressing complex challenges in civil engineering research and practice. Ebid's GP program, developed in 2004^31^, has been a pivotal tool, influencing diverse research fields and sparking numerous investigations. Widely adopted, it continues to shape computational methodologies, proving its efficacy in diverse scientific domains.

**Figure 9:**
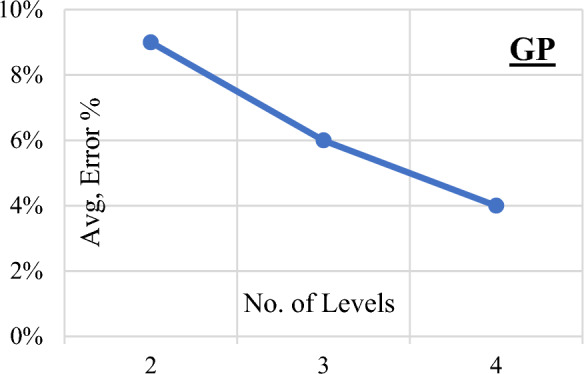
GP Model accuracy Vs complexity level.

**Figure 10:**
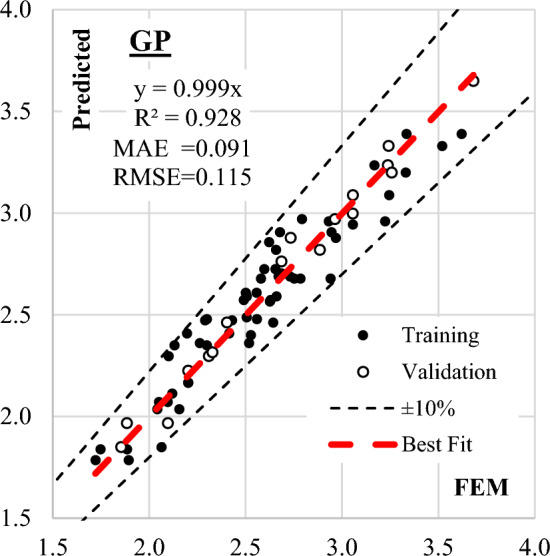
Relation between predicted and observed (qsh) values using the developed GP model.

Three Genetic Programming (GP) models were systematically developed, each characterized by varying levels of complexity ranging from two to four. The parameters governing the GP models, including population size, survivor size, and the number of generations, were set at 50,000, 15,000, and 1000, respectively. Figure 9 vividly illustrates the progressive enhancement in accuracy corresponding to the increasing complexity of the models. Equation (5) encapsulates the output formula for the total shear strength (qsh) derived from the first trial, as showcased in Fig. 10 depicting its fitness. The associated error percentage was recorded at a minimal 4.1%, underscoring the precision of the model, while the coefficient of determination (R2) stood impressively at 0.928. This indicates a high level of explanatory power in the model, affirming its reliability and effectiveness in predicting the shear strength of reinforced concrete beams.

5 $${\text{qsh}} = \frac{{\uprho^{\prime}}}{{\sqrt {\text{X}} - {\text{X}}}} + \frac{{\uprho^{\prime}}}{{\left( {\upmu .{\text{Fy}}} \right)^{1.5} - {\text{X}}}} + {\text{Ln}}\left( {{\text{Fcu}}{\text{.X}}^{0.44} - \upmu .{\text{Fy}}} \right)$$

where $$\text{X}=\frac{\text{d}.\upmu .\text{Fy}}{2.3\text{ S}}$$

#### (EPR) model

Evolutionary Polynomial Regression (EPR) is a specialized regression analysis technique that can be tailored for applications in civil engineering. In the realm of structural analysis and design, EPR is employed to model intricate relationships between key variables, offering a flexible and adaptive approach. Using a genetic algorithm, EPR evolves polynomial equations, allowing it to capture complex patterns in data relevant to civil engineering tasks. This method finds utility in developing predictive models for structural behavior, material properties, and other critical factors in research and practice. The integration of the Group Method of Data Handling (GMDH)^32^ with Evolutionary Polynomial Regression (EPR) enhances predictive modeling. GMDH autonomously identifies optimal model features and structure, providing relevant representations for EPR. This synergy captures complex non-linear patterns, offering a robust solution for regression tasks.

The three developed Evolutionary Polynomial Regression (EPR) models were limited to a six-level polynomial, resulting in a total of 924 possible terms, as represented by the following equation:

6 $${\text{Total}}\;{\text{ no}}.\;{\text{ of}}\;{\text{ terms }} = { 462 } + { 252 } + { 126 } + { 56 } + { 21 } + { 6 } + { 1 } = { 924}.$$

This intricate structure enables the models to capture complex relationships and interactions among the six inputs, striking a balance between model complexity and interpretability as follows:

7 $$\sum_{n=1}^{n=6}\sum_{m=1}^{m=6}\sum_{l=1}^{l=6}\sum_{k=1}^{k=6}\sum_{j=1}^{j=6}\sum_{i=1}^{i=6}{{{{{X}_{n}.X}_{m}.X}_{l}.X}_{k}.{X}_{j}.X}_{i}+\sum_{m=1}^{m=6}\sum_{l=1}^{l=6}\sum_{k=1}^{k=6}\sum_{j=1}^{j=6}\sum_{i=1}^{i=6}{{{{X}_{m}.X}_{l}.X}_{k}.{X}_{j}.X}_{i}+\sum_{l=1}^{l=6}\sum_{k=1}^{k=6}\sum_{j=1}^{j=6}\sum_{i=1}^{i=6}{{{X}_{l}.X}_{k}.{X}_{j}.X}_{i}+\sum_{k=1}^{k=6}\sum_{j=1}^{j=6}\sum_{i=1}^{i=6}{{X}_{k}.{X}_{j}.X}_{i}+\sum_{j=1}^{j=6}\sum_{i=1}^{i=6}{X}_{j}.{X}_{i}+\sum_{i=1}^{i=6}{X}_{i}+C$$

**Figure 11:**
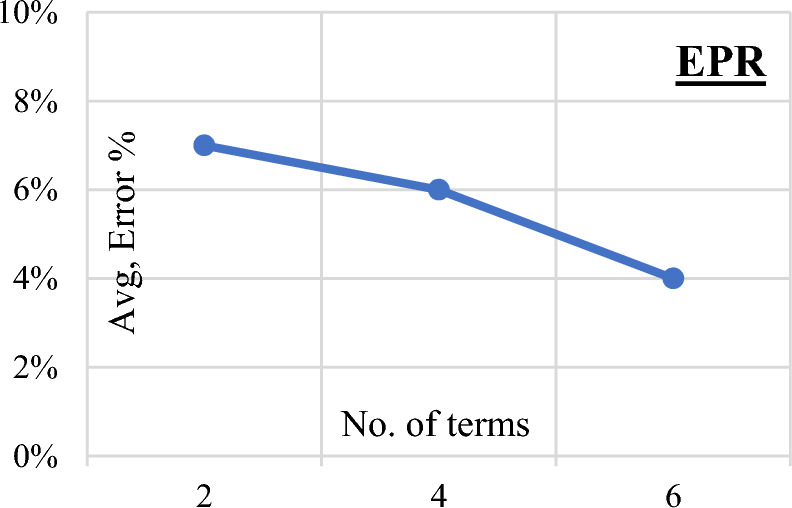
EPR Model accuracy Vs number of terms.

**Figure 12:**
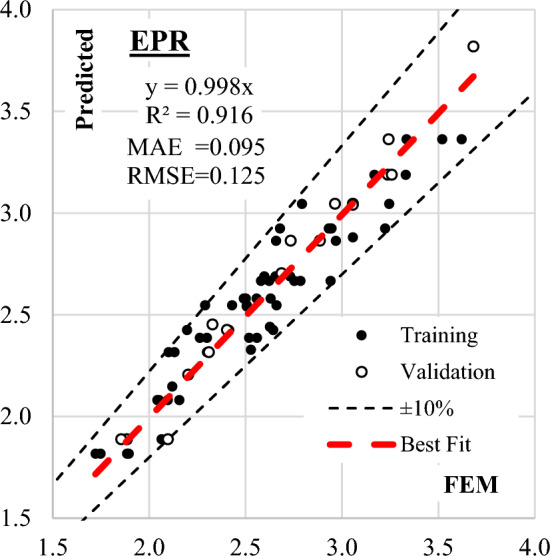
Relation between predicted and observed (qsh) values using the developed EPR model.

The Evolutionary Polynomial Regression (EPR) technique was employed to identify the most influential terms among the 924 possibilities for predicting (qsh). The iterative process began with only two terms and incrementally increased to six terms. As depicted in Fig. 11, fitness improved with the growing number of terms, reaching an optimal balance at six terms. Equation (8) represents the output of the final model, with its fitness shown in Fig. 12. The model exhibited an average error of 4.5% and an R^2^ value of 0.916, demonstrating its effectiveness in predicting shear strength values.

8 $$\text{qsh }=\frac{\text{Fcu}.{\text{S}}^{2}}{575{\text{d}}^{2}}+\frac{\text{S }\left[\text{Fcu}-128{\left(\upmu .\text{Fy}\right)}^{2}\right]}{35\text{d}}+\frac{1.25\text{d}}{\text{b}}-\frac{1.15\text{d}{\left(\upmu .\text{Fy}\right)}^{2}}{\text{S}} + 5.33{\left(\upmu .\text{Fy}\right)}^{2}+0.77$$

#### (ANN) model

Artificial Neural Networks (ANN) represents a cutting-edge computational approach extensively applied in civil engineering. In this field, ANN serves as a sophisticated machine learning technique capable of modeling complex relationships within structural systems. Mimicking the neural structure of the human brain, ANN processes vast amounts of data, learning and adapting to patterns to make predictions or classifications. Civil engineers harness the power of ANN for tasks such as predicting structural behavior, optimizing designs, and assessing the impact of various parameters on performance. Versatility and the ability to handle nonlinear relationships make ANN a valuable tool in civil engineering research, contributing to advancements in structural analysis, design, and decision support systems. The research employed SPSS Statistics^33^ to formulate the Artificial Neural Network (ANN) model.

**Figure 13:**
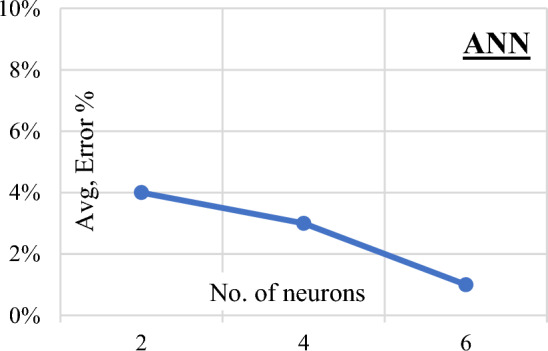
Average error percentage Vs. ANN layout**.**

**Figure 14:**
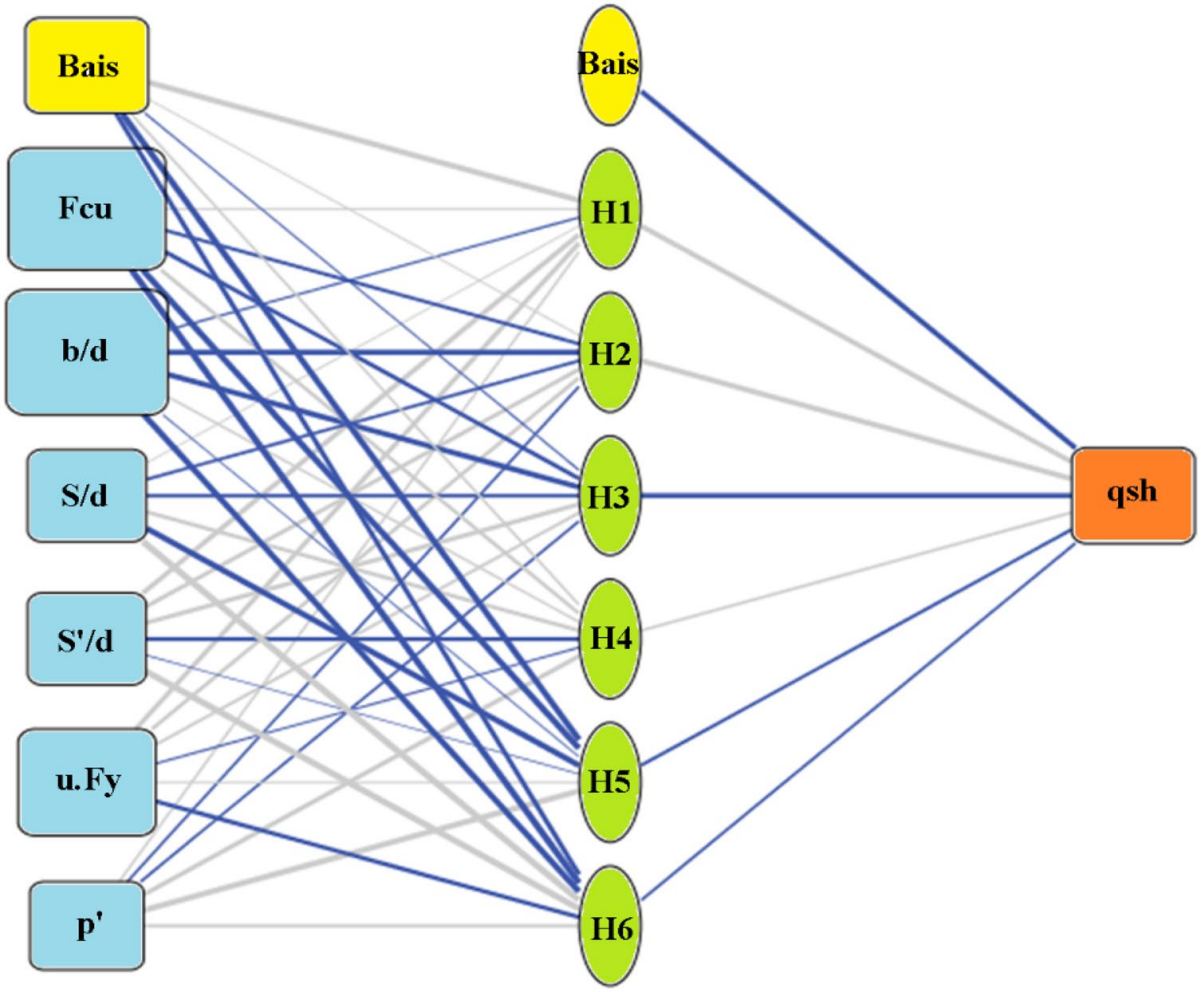
Layout for the developed ANN models.

**Figure 15:**
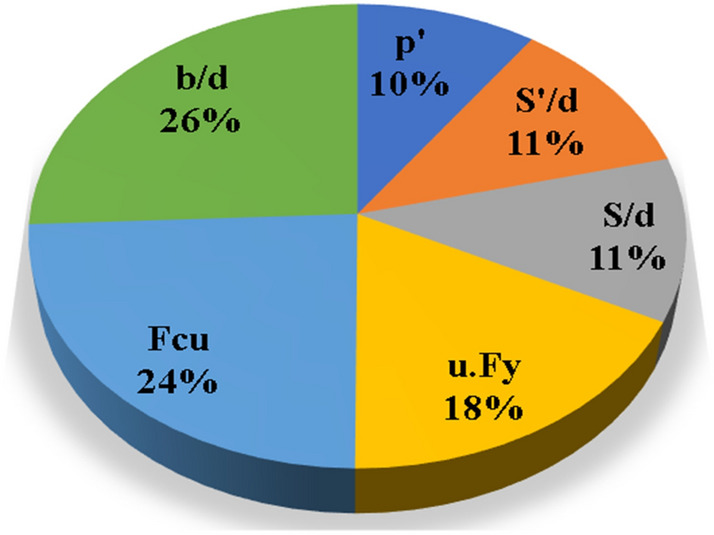
Relative importance of input parameters.

**Figure 16:**
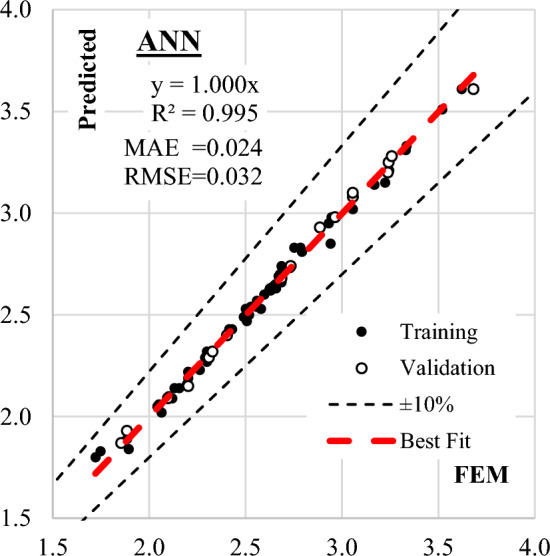
Relation between predicted and observed (qsh) values using the developed ANN model.

Three Artificial Neural Network (ANN) models were constructed to predict (qsh) values, employing normalization within the range of − 1.0–1.0 and Hyper Tan as the activation function. Backpropagation (BP) techniques were used for training, with the number of neurons in the hidden layer ranging from 2 to 6. The average error percentage depicted in Fig. 13 decreased as the number of neurons increased, with the last network layout presented in Fig. 14 and the weight matrix detailed in Table 4. The models exhibited an average error of 1.2% for the total dataset and an impressive (R^2^) value of 0.995, emphasizing their accuracy in predicting shear strength values. The relative importance values, illustrated in Fig. 15, highlighted that concrete strength and beam aspect ratio significantly influenced shear strength, followed by the amount of shear reinforcement and stirrups arrangement. The strong correlation between calculated and predicted values is demonstrated in Fig. 16.

**Table 4:** Weights matrix for the developed ANN.

	H(1)	H(2)	H(3)	H(4)	H(5)	H(6)	
(Bias)	3.65	0.03	− 0.04	0.15	− 4.10	− 1.29	
Fcu	0.07	− 0.59	− 0.82	0.64	− 4.16	− 5.94	
b/d	− 0.09	− 2.94	− 3.15	0.11	− 0.02	− 4.07	
S/d	0.03	− 0.39	− 0.57	0.69	− 3.98	7.12	
S'/d	1.41	0.75	0.97	− 0.70	− 0.01	4.51	
μ.Fy	1.07	0.67	0.32	− 0.06	0.05	− 0.83	
ρ'	0.12	− 0.15	− 0.22	1.02	3.82	0.51	

	H(1:1)	H(1:2)	H(1:3)	H(1:4)	H(1:5)	H(1:6)	(Bias)
qsh	2.46	3.07	− 2.75	0.29	− 0.41	− 0.26	− 2.81

#### Accuracy of the generated formulas

The accuracy of the developed models was meticulously assessed through a thorough evaluation of misclassified cases, expressed as the error percentage, by comparing the predicted and observed shear strength (qsh).

The superior predictive performance of Artificial Neural Network (ANN) in estimating shear strengths for wide-shallow reinforced concrete beams is attributed to its capability to capture complex and non-linear relationships within the dataset. The neural network's adeptness in learning and adapting during training, along with its automatic feature extraction, enhances its accuracy in representing the diverse shear behaviors of these beams. In contrast to Evolutionary Polynomial Regression (EPR) and Genetic Programming (GP), ANN's hierarchical structure allows it to identify subtle correlations, resulting in a more precise depiction of shear capacity. Overall, ANN's proficiency in handling the varied shear characteristics of wide-shallow beams can be ascribed to its adaptability, autonomous learning, and capability for extracting relevant features.

The similarity in predicting shear strengths for wide-shallow reinforced concrete beams between Evolutionary Polynomial Regression (EPR) and Genetic Programming (GP) can be attributed to their shared symbolic regression approach and the common utilization of polynomial expressions. Both methods employ genetic programming techniques to evolve mathematical equations fitting the dataset, leading to comparable outcomes. The emphasis on polynomial representations further contributes to the alignment in their predictive results. Overall, the close results between EPR and GP stem from their analogous approaches and the influence of dataset characteristics on their performance in estimating shear capacity for wide-shallow beams.

**Figure 17:**
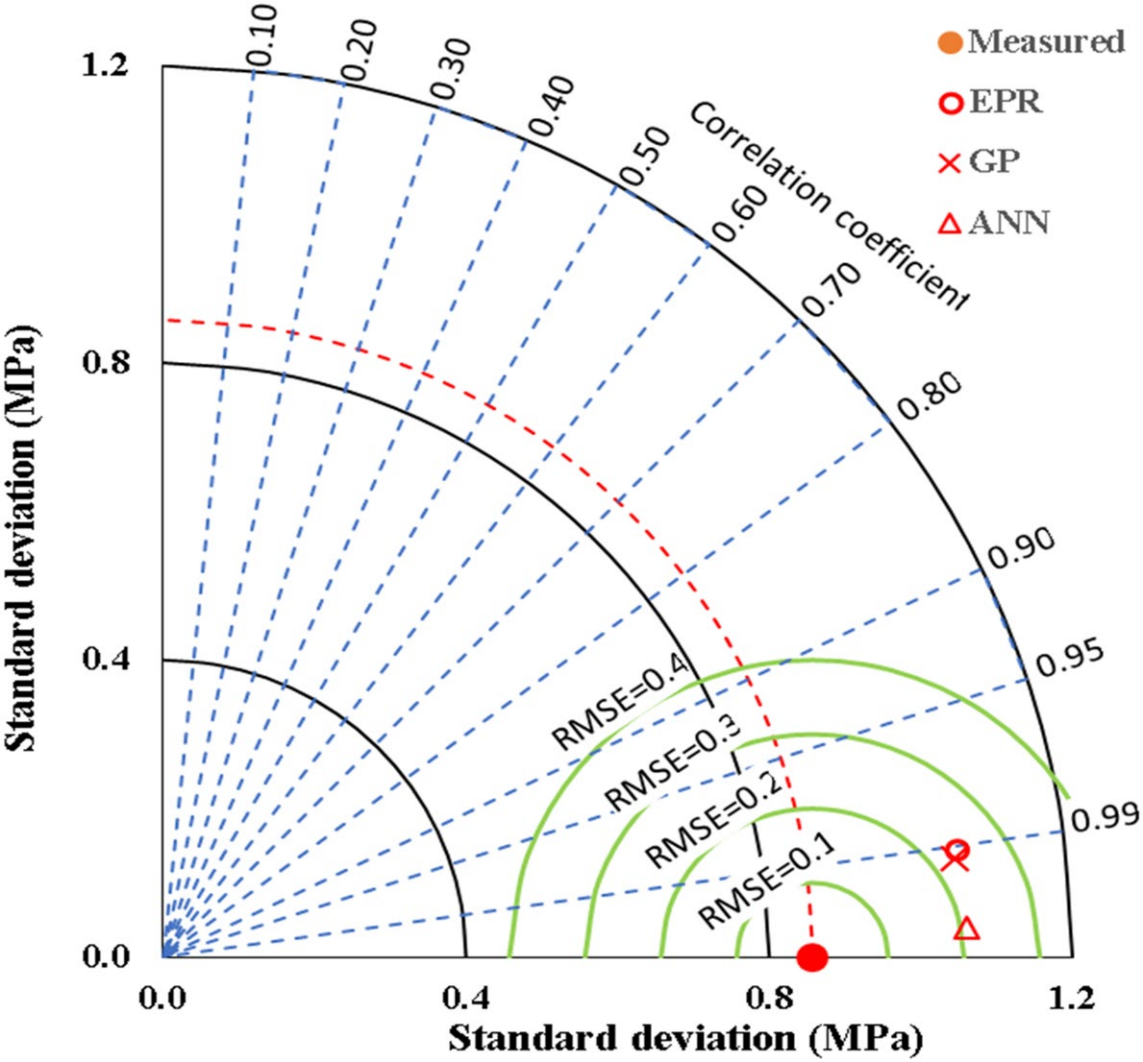
Comparing the accuracy of the developed models using Taylor charts.

The outcomes of this evaluation, encompassing all developed models, are succinctly summarized in Table 5 and Fig. 17. This comprehensive summary provides valuable conclusions about the effectiveness and precision of each model, facilitating a comparative analysis of their predictive performance against observed shear strength values.

**Table 5:** Accuracies of the developed models.

Technique	Model	SSE	MAE	MSE	RMSE	Error %	R^2^
GP	Equation (1)	1.02	0.091	0.013	0.115	4.1	0.928
EPR	Equation (3)	1.19	0.095	0.016	0.125	4.5	0.916
ANN	Equation (7)	0.08	0.024	0.001	0.032	1.2	0.995

## Conclusions

This research endeavors to forecast the total shear stress at failure of the wide reinforced concrete beams, incorporating material strength parameters, geometrical configuration, and rebar arrangements, employing three machine learning-based techniques. The study comprehensively evaluates and compares the accuracy of these developed models. The conclusions serve to illuminate the efficacy and reliability of each predictive model in estimating the total shear stress at failure of the wide reinforced concrete beams as follows:

- The proposed finite element model was verified, showing differences in loads and deflections of up to 8% and 12%, respectively.
- Genetic Programming (GP) and Evolutionary Polynomial Regression (EPR) models demonstrated excellent accuracy, with a correlation coefficient exceeding 99%. GP exhibited a slightly superior accuracy of (96%) compared to EPR (95.5%).
- Conversely, the Artificial Neural Network (ANN) model showcased the highest accuracy (99%), and a nearly perfect correlation coefficient, but had higher complexity.
- Concrete strength (Fcu) and beam aspect ratio (b/d) exerted the most substantial influence on total shear strength, followed by the amount of shear reinforcement (μ.Fy), and the stirrup arrangement (S/d, S^′^/d).
- The validity of the developed models is confined to the considered range of parameter values; for predictions beyond this range, further verification is recommended.

### Supplementary Information

Supplementary Information.

## Data Availability

All data is introduced and discussed through the article.

## Abbreviations

Fcu: Characteristic cube compressive strength at 28 days (MPa),
b/d: The aspect ratio of the beam (width/depth),
S/d: Longitudinal stirrups spacing/beam depth,
S^′^/d: Transvers stirrups spacing/beam depth,
μ.Fy: Shear reinforcement ratio (Ash/b.S) x yield stress of the stirrups,
ρ^′^: Compression rebars ratio (As’/b.d)
qsh: Total shear stress at failure (Q/b.d)
