# Supplementary material for: Predictive modeling of wide-shallow RC beams shear strength considering stirrups effect using (FEM-ML) approach
Source: Sci Rep. 2024 May 31;14:12523. doi: 10.1038/s41598-024-62532-y (PMC11143311; doi:10.1038/s41598-024-62532-y)
Supplement: Supplementary file 1 — Supplementary Information. [file 41598_2024_62532_MOESM1_ESM.docx]

**Appendix**

| Training data set | | | | | | |
| --- | --- | --- | --- | --- | --- | --- |
| Fcu (MPa) | **b/d** | **S/d** | **S'/d** | **u.Fys (MPa)** | **p'** | **qsh (MPa)** |
| 45.00 | 1.67 | 0.28 | 0.67 | 0.46 | 0.02 | 2.93 |
| 45.00 | 5.00 | 0.28 | 0.67 | 0.46 | 0.01 | 2.19 |
| 35.00 | 3.33 | 0.56 | 0.67 | 0.79 | 0.01 | 2.94 |
| 35.00 | 3.33 | 1.11 | 1.11 | 0.79 | 0.01 | 2.63 |
| 25.00 | 1.67 | 0.28 | 1.67 | 0.46 | 0.02 | 2.13 |
| 25.00 | 1.67 | 0.28 | 1.67 | 1.25 | 0.02 | 2.49 |
| 45.00 | 5.00 | 0.28 | 5.00 | 0.46 | 0.02 | 2.64 |
| 25.00 | 1.67 | 1.11 | 1.67 | 0.46 | 0.01 | 2.52 |
| 45.00 | 1.67 | 1.11 | 0.67 | 1.25 | 0.01 | 3.52 |
| 25.00 | 1.67 | 0.28 | 1.67 | 1.25 | 0.01 | 2.50 |
| 45.00 | 5.00 | 0.28 | 5.00 | 1.25 | 0.02 | 2.67 |
| 35.00 | 3.33 | 0.56 | 1.11 | 0.46 | 0.01 | 2.12 |
| 35.00 | 3.33 | 0.56 | 1.11 | 0.79 | 0.02 | 2.62 |
| 25.00 | 5.00 | 1.11 | 5.00 | 1.25 | 0.02 | 2.20 |
| 45.00 | 5.00 | 1.11 | 0.67 | 0.46 | 0.02 | 2.66 |
| 45.00 | 1.67 | 0.28 | 1.67 | 1.25 | 0.01 | 3.17 |
| 45.00 | 1.67 | 1.11 | 0.67 | 0.46 | 0.02 | 3.24 |
| 45.00 | 5.00 | 1.11 | 5.00 | 0.46 | 0.01 | 2.29 |
| 25.00 | 5.00 | 1.11 | 0.67 | 1.25 | 0.01 | 2.20 |
| 25.00 | 5.00 | 0.28 | 0.67 | 0.46 | 0.02 | 1.88 |
| 45.00 | 5.00 | 0.28 | 5.00 | 1.25 | 0.01 | 2.60 |
| 45.00 | 5.00 | 0.28 | 0.67 | 1.25 | 0.02 | 2.73 |
| 45.00 | 1.67 | 1.11 | 1.67 | 1.25 | 0.02 | 3.33 |
| 45.00 | 5.00 | 0.28 | 5.00 | 0.46 | 0.01 | 2.41 |
| 35.00 | 3.33 | 0.56 | 3.33 | 0.79 | 0.01 | 2.58 |
| 45.00 | 5.00 | 1.11 | 5.00 | 0.46 | 0.02 | 2.50 |
| 45.00 | 5.00 | 1.11 | 5.00 | 1.25 | 0.01 | 2.66 |
| 25.00 | 5.00 | 0.28 | 5.00 | 1.25 | 0.02 | 2.04 |
| 25.00 | 5.00 | 0.28 | 5.00 | 1.25 | 0.01 | 2.05 |
| 45.00 | 1.67 | 1.11 | 0.67 | 1.25 | 0.02 | 3.62 |
| 25.00 | 5.00 | 0.28 | 0.67 | 1.25 | 0.02 | 2.15 |
| 35.00 | 3.33 | 0.56 | 1.11 | 0.79 | 0.01 | 2.78 |
| 35.00 | 5.00 | 0.56 | 1.11 | 0.79 | 0.01 | 2.50 |
| 25.00 | 5.00 | 0.28 | 5.00 | 0.46 | 0.02 | 1.75 |
| 25.00 | 1.67 | 0.28 | 0.67 | 1.25 | 0.02 | 2.63 |
| 45.00 | 3.33 | 0.56 | 1.11 | 0.79 | 0.01 | 3.05 |
| 45.00 | 5.00 | 0.28 | 0.67 | 1.25 | 0.01 | 2.65 |
| 25.00 | 1.67 | 0.28 | 0.67 | 0.46 | 0.01 | 2.10 |
| 25.00 | 1.67 | 1.11 | 1.67 | 0.46 | 0.02 | 2.56 |
| 25.00 | 1.67 | 0.28 | 0.67 | 1.25 | 0.01 | 2.56 |
| 25.00 | 1.67 | 1.11 | 0.67 | 1.25 | 0.01 | 2.69 |
| 45.00 | 1.67 | 0.28 | 0.67 | 1.25 | 0.02 | 3.33 |
| 25.00 | 5.00 | 1.11 | 5.00 | 1.25 | 0.01 | 2.20 |
| 25.00 | 5.00 | 0.28 | 0.67 | 0.46 | 0.01 | 1.72 |
| 35.00 | 3.33 | 0.56 | 1.11 | 0.79 | 0.01 | 2.75 |
| 45.00 | 1.67 | 1.11 | 1.67 | 0.46 | 0.01 | 2.79 |
| 25.00 | 5.00 | 1.11 | 5.00 | 0.46 | 0.01 | 2.06 |
| 25.00 | 1.67 | 1.11 | 0.67 | 0.46 | 0.02 | 2.30 |
| 25.00 | 1.67 | 0.28 | 0.67 | 0.46 | 0.02 | 2.30 |
| 25.00 | 1.67 | 1.11 | 1.67 | 1.25 | 0.01 | 2.68 |
| 45.00 | 5.00 | 1.11 | 0.67 | 0.46 | 0.01 | 2.43 |
| 45.00 | 1.67 | 0.28 | 1.67 | 0.46 | 0.01 | 2.94 |
| 25.00 | 5.00 | 0.28 | 0.67 | 1.25 | 0.01 | 2.09 |
| 45.00 | 1.67 | 0.28 | 1.67 | 0.46 | 0.02 | 3.22 |
| 45.00 | 5.00 | 1.11 | 0.67 | 1.25 | 0.02 | 2.97 |
| 45.00 | 1.67 | 0.28 | 0.67 | 0.46 | 0.01 | 2.68 |
| 25.00 | 5.00 | 0.28 | 5.00 | 0.46 | 0.01 | 1.89 |
| 25.00 | 1.67 | 1.11 | 0.67 | 0.46 | 0.01 | 2.26 |
| 35.00 | 3.33 | 0.28 | 1.11 | 0.79 | 0.01 | 2.53 |
| 25.00 | 1.67 | 1.11 | 0.67 | 1.25 | 0.02 | 2.69 |
| 25.00 | 5.00 | 1.11 | 0.67 | 1.25 | 0.02 | 2.20 |
| 35.00 | 3.33 | 0.56 | 1.11 | 1.25 | 0.01 | 3.68 |
| 45.00 | 1.67 | 1.11 | 0.67 | 0.46 | 0.01 | 2.96 |
| 25.00 | 5.00 | 1.11 | 5.00 | 0.46 | 0.02 | 2.10 |
| 45.00 | 1.67 | 1.11 | 1.67 | 1.25 | 0.01 | 3.24 |
| 25.00 | 1.67 | 0.28 | 1.67 | 0.46 | 0.01 | 2.31 |
| 45.00 | 1.67 | 0.28 | 0.67 | 1.25 | 0.01 | 3.24 |
| 45.00 | 5.00 | 1.11 | 5.00 | 1.25 | 0.02 | 2.73 |
| 45.00 | 1.67 | 1.11 | 1.67 | 0.46 | 0.02 | 3.06 |
| 45.00 | 5.00 | 1.11 | 0.67 | 1.25 | 0.01 | 2.88 |
| 25.00 | 3.33 | 0.56 | 1.11 | 0.79 | 0.01 | 2.33 |
| 35.00 | 1.67 | 0.56 | 1.11 | 0.79 | 0.01 | 3.06 |
| 45.00 | 1.67 | 0.28 | 1.67 | 1.25 | 0.02 | 3.26 |
| 45.00 | 5.00 | 0.28 | 0.67 | 0.46 | 0.02 | 2.40 |
| 25.00 | 5.00 | 1.11 | 0.67 | 0.46 | 0.02 | 1.88 |
| 25.00 | 1.67 | 1.11 | 1.67 | 1.25 | 0.02 | 2.69 |
| 25.00 | 5.00 | 1.11 | 0.67 | 0.46 | 0.01 | 1.85 |
